# The influence of clinical, environmental, and socioeconomic factors on five-year patient survival after kidney transplantation

**DOI:** 10.1590/2175-8239-JBN-3865

**Published:** 2018-06-04

**Authors:** Priscila Ruppel, Claudia R. Felipe, Jose O. Medina-Pestana, Liliane Lumi Hiramoto, Laila Viana, Alexandra Ferreira, Wilson Aguiar, Mayara Ivani, Adrieli Bessa, Marina Cristelli, Melissa Gaspar, Helio Tedesco-Silva

**Affiliations:** 1 Universidade Federal de São Paulo, São Paulo, SP, Brasil.

**Keywords:** Kidney Transplantation, Mortality, Risk Factors, Socioeconomic Factors, Transplante de Rim, Mortalidade, Fatores de Risco, Fatores Socioeconômicos

## Abstract

**Introduction:**

The risk of death after kidney transplant is associated with the age of the recipient, presence of comorbidities, socioeconomic status, local environmental characteristics and access to health care.

**Objective:**

To investigate the causes and risk factors associated with death during the first 5 years after kidney transplantation.

**Methods:**

This was a single-center, retrospective, matched case-control study.

**Results:**

Using a consecutive cohort of 1,873 kidney transplant recipients from January 1^st^ 2007 to December 31^st^ 2009, there were 162 deaths (case group), corresponding to 5-year patient survival of 91.4%. Of these deaths, 25% occurred during the first 3 months after transplant. The most prevalent cause of death was infectious (53%) followed by cardiovascular (24%). Risk factors associated with death were history of diabetes, dialysis type and time, unemployment, delayed graft function, number of visits to center, number of hospitalizations, and duration of hospital stay. After multivariate analysis, only time on dialysis, number of visits to center, and days in hospital were still associated with death. Patients who died had a non-significant higher number of treated acute rejection episodes (38% *vs*. 29%, *p* = 0.078), higher mean number of adverse events per patient (5.1 ± 3.8 *vs*. 3.8 ± 2.9, *p* = 0.194), and lower mean eGFR at 3 months (50.8 ± 25.1 *vs*. 56.7 ± 20.7, *p* = 0.137) and 48 months (45.9 ± 23.8 *vs*. 58.5 ± 20.2, *p* = 0.368).

**Conclusion:**

This analysis confirmed that in this population, infection is the leading cause of mortality over the first 5 years after kidney transplantation. Several demographic and socioeconomic risk factors were associated with death, most of which are not readily modifiable.

## RESUMO

**Introdução:**

O risco de óbito após transplante renal está associado à idade do receptor, presença de comorbidades, condição socioeconômica, às características ambientais locais e ao acesso a serviços de atenção à saúde.

**Objetivo:**

Investigar as causas e fatores de risco associados ao óbito nos primeiros cinco anos após o transplante renal.

**Métodos:**

Este é um estudo unicêntrico retrospectivo com pareamento dos grupos caso e controle.

**Resultados:**

Em uma coorte consecutiva de 1.873 receptores de transplante renal atendidos de 1/1/2007 a 31/12/2009 foram registrados 162 óbitos (grupo caso), correspondendo a uma taxa de sobrevida após cinco anos de 91,4%. Dos óbitos registrados, 25% ocorreram nos primeiros três meses após o transplante. A causa de óbito mais prevalente foi infecção (53%), seguida de doença cardiovascular (24%). Os fatores de risco associados a mortalidade foram histórico de diabetes, tipo e tempo em diálise, desemprego, função tardia do enxerto, número de consultas, número de hospitalizações e tempo de internação hospitalar. Após análise multivariada, apenas o tempo em diálise, o número de consultas e dias de internação permaneceram associados a mortalidade. Os pacientes que foram a óbito tiveram um número não significativamente maior de tratamentos de episódios de rejeição aguda (38% *vs*. 29%; *p* = 0,078), maior número médio de eventos adversos por paciente (5,1 ± 3,8 *vs*. 3,8 ± 2,9; *p* = 0,194) e TFGe média mais baixa aos três meses (50,8 ± 25,1 *vs*. 56,7 ± 20,7; *p* = 0,137) e 48 meses (45,9 ± 23,8 *vs*. 58,5 ± 20,2; *p* = 0,368).

**Conclusão:**

A presente análise confirmou que nessa população, a infecção foi a principal causa de mortalidade nos primeiros cinco anos após transplante renal. Vários fatores de risco demográficos e socioeconômicos foram associados a mortalidade, a maioria não prontamente modificável.

## INTRODUCTION

Death with functioning graft is a major cause of graft loss among kidney transplant recipients worldwide. A recent review of almost ten thousand kidney transplants revealed that death with functioning graft accounted for 45% of kidney graft losses.^1^ Contrary to developed countries, where cardiovascular events are the major cause of death, in this large cohort of kidney transplant recipients from a developing country, the major cause of death was infection, not only during the first year but during any time after kidney transplantation.^1^ The risk of death increases with the age of the recipient from 5.8% under 50 years to 45.5% in patients older than 80 years. The risk of death increases even more in the presence of comorbidities such as hypertension, dyslipidemia, and post-transplant diabetes.^2^

A previous study revealed several risk factors associated with death within the first 6 months after transplantation, including donor age and cause of death, recipient gender, HLA compatibility, changes in electrocardiogram, weight at the time of transplantation, financial assistance, monthly income, and having children and family support.^3^ Patients living in lower socioeconomic areas have a higher risk of death^2^ and patients with lower income presented an additional 36.2% risk for graft loss.^4^ In the past, evaluating the impact of socioeconomic factors on the outcome of transplantation was difficult, so race was used as a surrogate for patient's socioeconomic status. In that scenario, Black patients, with usually worse socioeconomic characteristics, had lower graft survival.^5^ Socioeconomic variables have always influenced health-related outcomes.^3^ In a previous study, four of ten variables influencing transplant outcomes were socioeconomic, perhaps explaining the apparent discrepancy in the cause of death between developed and developing countries.^3^

Other socioeconomic factors have been associated with graft loss and death after renal transplant. One of these factors is the human development index (HDI), a statistical measure used as an indicator of health to classify regions considering life expectancy, education, and per capita income.^6^^,^ 7 Finally, local environment also influence health-related outcomes. As such, sanitation, weather, endemic diseases, and access to health care may influence kidney transplant outcomes. Considering this complex scenario, we investigated further the causes and risk factors associated with death over the first five years after kidney transplantation.

## METHODS

### STUDY DESIGN

This was a single-center, retrospective, case-control study comparing demographical and clinical outcomes between patients who died and paired matched living controls during the first 5 years after kidney transplantation. The case-control design hindered several traditional risk factors such as recipient age, diabetes, and cardiovascular disease. However, the aim was to investigate beyond these traditional risk factors and determine whether local socioeconomic and environmental risk factors would be involved. The data was extracted from the electronic database and judged accordingly. The study was approved by the local ethics committee.

### POPULATION

We included only patients who received a kidney transplant from January 1, 2007 to December 31, 2009, thus allowing 5 years of follow up by December 31, 2014. During this period 2305 kidney transplants were performed. We excluded 140 recipients of retransplants, 126 recipients of combined kidney/pancreas transplants and 166 pediatric recipients. Of the final cohort of 1,873 patients, we identified all deaths within the first 5 years after transplantation to form the case group. The control group (1:1) was selected from the same cohort by matching the following variables: date of transplant, recipient age (+/- 5 years), gender and race, donor age (+/- 5 years), gender and type (living or deceased) and use of thymoglobulin induction.

### OBJECTIVE

The objective of this study was to identify risk factors associated with death during the first 5 years after kidney transplantation. We also analyzed the socioeconomic and demographic characteristics, incidence of hospitalizations, renal function, and specific causes of death.

### DEMOGRAPHIC AND SOCIOECONOMIC VARIABLES

Data were collected retrospectively from medical records and included recipient, donor, and transplant-related variables. We also assessed human development index (HDI) of the city of each patient using the Human Atlas of Human Development (http://www.atlasbrasil.org.br/, assessed on 13 of June, 2016)^7^ and travel distance to the transplant center using Google Maps(tm) (maps.google.com). Professions were classified in three main categories based on the information obtained at the time of transplantation: higher occupations (high-level hierarchical position), intermediate occupations (lower hierarchical rank) and lower occupations (manual or routine labor), also including those who had never worked or were unemployed according the National Socio-economic Classification (NS-SEC).^8^

### IMMUNOSUPPRESSION AND PROPHYLAXIS

The use of induction therapy, with basiliximab or rabbit anti-thymocyte globulin, and the maintenance immunosuppressive regimens consisted primarily of a calcineurin inhibitor in combination with an anti-proliferative drug or an mTOR inhibitor and were based on institutional protocol derived from evaluation of immunological risk. All patients received corticosteroids, 1 mg intravenous bolus of methylprednisolone before graft revascularization followed by 0.5 mg/kg/day of prednisone with a taper to 5 mg/day between 30 to 45 days after transplantation. All patients received sulphametaxasol trimetropin for at least 6 months for prophylaxis against *Pneumocystis jirovecii* pneumonia and urinary tract infection. All patients received albendazole for parasitic infections. None of the patients received pharmacological prophylaxis for cytomegalovirus (CMV) infection. Instead, preemptive treatment was performed in patients deemed as high risk for developing CMV infection: (1) seronegative CMV kidney transplant recipients from seropositive CMV donors (D+/R-); (2) use of r-ATG for induction and (3) use of MPS for maintenance therapy; (4) after treatment of acute rejection episodes.

### CLINICAL PARAMETERS

Delayed graft function (DGF) was defined as the need for dialysis during the first week after transplantation, except for one dialysis due to hyperkalemia. Estimated glomerular filtration rate (eGFR) was calculated using the MDRD formula. Acute rejection episodes included biopsy-proven acute rejection (BPAR) (Banff 2005) and clinical acute rejections were episodes of acute graft dysfunction treated with methylprednisolone for at least 3 days without histological confirmation (no biopsy, biopsy with insufficient representation of renal compartments or biopsy without evidence of acute rejection). All causes of death and graft loss were assessed. Patients transferred to another center or those with missing appointments for more than 6 months were considered lost to follow-up.

### OUTPATIENT VISITS AND HOSPITALIZATIONS

The number of outpatient visits and hospital readmission days were calculated in both groups during the follow-up time in months. All serious adverse events (SAE) during each hospitalization were captured and classified according to the Common Terminology Criteria for Adverse Events (CTCAE) version 4.0.

### STATISTICAL ANALYSIS

The Kolmogorov-Smirnov test was performed to verify the normality of the numerical variables. Variables with normal distribution were summarized by mean and standard deviation and differences compared using the Student's *t*-test. Variables with non-normal distribution were summarized by median and range and the differences were compared using the non-parametric U Mann-Whitney test. Frequencies and the chi-square test were used for qualitative variables. Uni- and multivariable risk analysis was performed using Cox regression and 95% confidence intervals. All tests were analyzed using the SPSS Statistics 18.0 program (SPSS Inc., Chicago, IL). Values of *p* < 0.05 were reported as statistically significant.

## RESULTS

### POPULATION

Of 1873 adult recipients of first kidney transplants, 162 died, 159 had graft loss and 165 were lost to follow up 5 years after transplantation. Corresponding 5-year graft and death-censored survivals were 91.4%, 82.9% and 90.7%, respectively. The 162 deaths were matched to 144 controls using the predefined criteria and small deviations were necessary for the remaining 18 controls (transplants prior to 2007 [n = 4] or after 2009 [n = 5], without matched donor age [n = 3], gender [n = 4] or donor type [n = 2]). Among the 162 control cases, there were 9 graft losses, 11 losses to follow-up and 5 transplants after 2009, yielding 137 patients who completed 5 years of follow-up.

### DEMOGRAPHY

Patients who died during the first 5 years after transplantation were more likely to have *diabetes mellitus*, were on dialysis for a longer period of time and three of them had prior contact with tuberculosis (Table 1). There was no difference in marital status, religion, and HDI. Patients who died tended to have lower level of education and to be unemployed. Interestingly, patients who died lived closer to the transplant center (Table 2). There was no evident difference in use and type of induction agent or the maintenance of immunosuppressive regimens. The majority of patients received induction therapy followed by tacrolimus with mycophenolate or azathioprine (Table 3).

**Table 1:** Demographics characteristics of the study population

Variables	death (n = 162)	control (n = 162)	*p*
Recipient age (years), mean ± SD	50.3 ± 12.2	49.8 ± 12.6	0.971
Recipient gender (male), N (%)	96 (59.3)	102 (63)	0.494
Cause of chronic kidney disease, N (%)			0.418
Undetermined	75 (46.3)	79 (48.8)	
Hypertension	14 (8.6)	11 (6.8)	
*Diabetes mellitus*	32 (19.8)	24 (14.8)	
Glomerulonephritis	11 (6.8)	17 (10.5)	
Time on dialysis (months), mean ± SD	53.9 ± 41.5	36.9 ± 31.0	< 0.001
Type of renal replacement therapy, N (%)			0.019
Preemptive	2 (1.2)	12 (7.4)	
Hemodialysis	146 (90.1)	140 (86.4)	
Peritoneal	14 (8.7)	10 (6.2)	
History of *diabetes mellitus*, N (%)	47 (29)	30 (18.5)	0.026
Prior contact with tuberculosis, N (%)	3 (1.9)	0 (0)	0.082
Panel reactive antibodies, (%)			
Class I, mean ± SD	7 ± 17	8 ± 20	0,265
Class II, mean ± SD	6 ± 19	3 ± 13	0,01
HLA mismatches, mean ± SD	2.8 ± 1.6	2.3 ± 1.6	0.64
Donor age, years, mean ± SD	46.5 ± 12.7	46.0 ± 12.9	0.763
Donor gender, male, N (%)	79 (48.7)	87 (53.7)	0.405
Donor type, N (%)			0.968
Living	51 (31.5)	53(32.7)	
Deceased Standard criteria	76 (46.9)	74 (45.7)	
Deceased Expanded Criteria	35 (21.6)	35 (21.6)	
Deceased donor cold ischemia time, hours, mean ± SD	25.4 ± 6.42	24.9 ± 5.76	0.163

HLA: human leukocyte.

**Table 2:** Socioeconomic and cultural characteristics of the study population

Variables, N (%)	death (N=162)	control (N=162)	*p*
Marital status			0.966
Married	104 (64.2)	104 (64.2)	
Cohabitation	3 (1.9)	5 (3.1)	
Separated	2 (1.2)	3 (1.9)	
Divorced	6 (3.7)	7 (4.3)	
Not married	37 (22.8)	33 (20.4)	
Widower	8 (4.9)	9 (5.6)	
Others	2 (1.2)	1 (0.6)	
Religion			0.255
Adventist	1 (0.6)	0 (0)	
Atheist	6 (3.7)	9 (5.6)	
Batista	0 (0)	2 (1.2)	
Catholic	109 (67.3)	96 (59.3)	
Evangelical	27 (16.7)	33 (20.4)	
Jehovah's Witness	3 (1.9)	1 (0.6)	
Protestant	0 (0)	3 (1.9)	
Spiritist	5 (3.1)	3 (1.9)	
Others	11 (6.8)	15 (9.3)	
Degree of instruction			0.133
Primary incomplete	29 (17.9)	22 (13.6)	
Secondary incomplete	82 (50.6)	72 (44.4)	
Secondary or higher	51 (31.5)	68 (42.0)	
Profession classification			< 0.001
Intermediate	8 (4.9)	31 (19.1)	
Lower	75 (46.3)	80 (49.4)	
Unemployed	79 (48.8)	51 (31.5)	
Human Development Index of the city-2010			0.373
Very high	68 (42)	72 (44.4)	
High	88 (54.3)	87 (53.7)	
Medium	6 (3.7)	2 (1.2)	
Low	0 (0)	1 (0.6)	
Travel distance to center, Km, mean ± SD	93.98 ± 191.87	144.92 ± 342.93	0.011

**Table 3:** Initial immunosuppression

Regimen, n (%)	death (n = 162)	control (n = 162)
Induction		
none	52 (32)	65 (40)
basiliximab	96 (59)	80 (49)
anti-thymocyte globulin	14 (9)	17 (11)
Maintenance		
Tacrolimus/mycophenolate	78 (48)	68 (42)
Tacrolimus/azathioprine	54 (33)	56 (35)
Tacrolimus/ mTOR inhibitor	2 (1)	3 (2)
Cyclosporine/mycophenolate	7 (4)	6 (4)
Cyclosporine/azathioprine	3 (2)	13 (8)
Cyclosporine/mTOR inhibitor	5 (3)	4 (3)
Other	13 (8)	12 (8)

mTOR: mammalian target of rapamycin.

### CLINICAL OUTCOMES

Patients who died had a higher incidence of delayed graft function, higher incidence of treated acute rejection episodes, higher number of acute rejection episodes treated with rabbit anti-thymocyte globulin (r-ATG), and lower eGFR compared to the control group during the 5 years of follow up (Table 4).

**Table 4:** Clinical outcomes during the 5-year follow-up

	Death	Control	*p*
	(N=162)	(N=162)	
Delayed graft function, n (%)	67 (41)	47 (29)	0.012
Treatment for acute rejection, n (%)	62 (38)	47 (29)	0.078
All acute rejection treated with r-ATG, n (%)	22	11	
eGRF, mean ± DP (n)			
Day 1	12.3 ± 11.6 (161)	13.3 ± 12.1 (162)	0.307
Month 3	50.8 ± 25.1 (118)	56.7 ± 20.7 (157)	0.137
Month 6	50.8 ± 21.6 (102)	58.4 ± 20.8 (154)	0.839
Month 12	55.8 ± 25.3 (91)	61.4 ± 20.4 (148)	0.1
Month 24	49.9 ± 21.9 (67)	59.8 ± 20.5 (148)	0.669
Month 36	50.3 ± 23.8 (43)	60.2 ± 20.3 (141)	0.162
Month 48	45.9 ± 23.8 (20)	58.5 ± 20.2 (137)	0.368
Month 60	-	58.1 ± 21.3 (137)	

r-ATG: rabbit anti-thymocyte globulin; eGRF: estimated glomerular filtration rate using the modification of diet in renal disease formula (mL/min/1.73 m^2^).

### VISITS, HOSPITALIZATIONS, AND SERIOUS ADVERSE EVENTS

**Figure 1:**
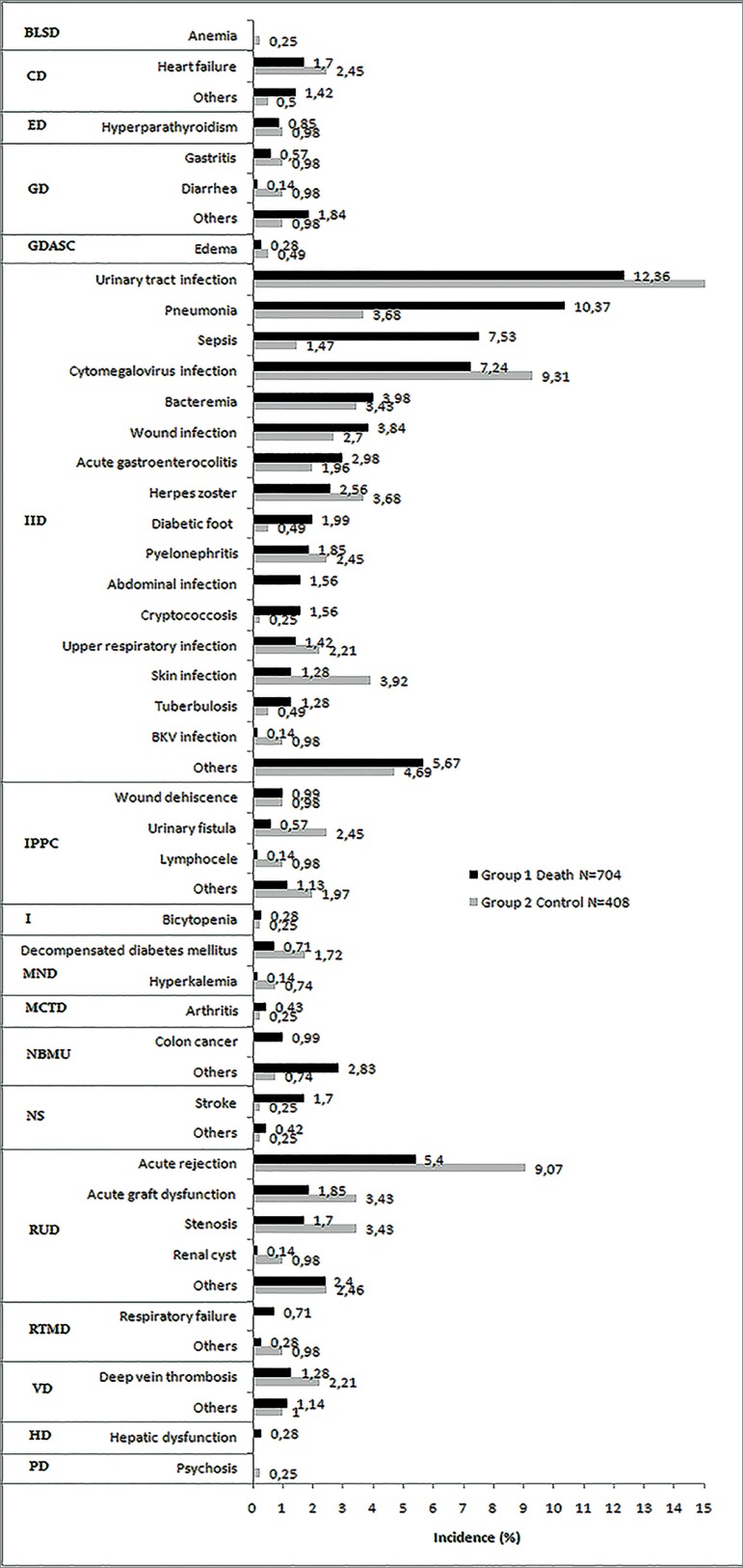
Causes of adverse events according to CTCAE. BLSD: blood and lymphatic system disorders; CD: Cardiac disorders; ED: endocrine disorders; GD: gastrointestinal disorders; GDASC: general disorders and administration site condition; IID: infections and infestation disorders; IPPC: injury, poisoning and procedural complications; I: investigation; MND: metabolism and nutrition disorders; MCTD: musculoskeletal and connective tissue disorder; NBMU: neoplasms benign, malignant and unspecified (including cysts and polyps); NS: nervous system; RUD: renal and urinary disorders; RTMD: respiratory, thoracic and mediastinal disorders; VD: vascular disorder; HD: hepatobiliary disorders; PD: psychiatric disorders.

Patients who died had a higher number of visits to the transplant center, hospitalizations, days in the hospital, and adverse events (Table 5). Infections/infestations accounted for the majority of the adverse events. Urinary tract infection, pneumonia, sepsis, and CMV infection accounted for the majority of infections leading to hospital readmissions. While pneumonia and sepsis were more prevalent among patients who died, no clear differences were observed for urinary tract and CMV infections. Interestingly, skin infection, urinary fistula, and acute rejection were more prevalent in the control group, with no significant differences in other specific adverse events during hospitalizations comparing both groups (Figure 1).

**Table 5:** Visits, hospitalizations, and adverse events during the 5 years of follow up

	death (N = 162)	control (N = 162)	*p*
Visits to the transplant center, n/month, mean ± SD	1.5 ± 1.3	0.8 ± 0.4	< 0.001
Number of patients hospitalized, N (%)	139 (86)	107 (66)	< 0.001
Rehospitalizations, N	446	294	
Rehospitalizations per patient, mean ± SD	2.7 ± 2.7	1.8 ± 2.1	0.058
Days in hospital (n/month), mean ± SD	12.7 ± 32.7	2.8 ± 16.3	< 0.001
Adverse events, N	704	408	
Adverse events per patient, mean ± SD	5.1 ± 3.8	3.8 ± 2.9	0.194

### RISK FACTORS AND CAUSES OF DEATH

Overall, infection was the main cause of death followed by cardiovascular events (Table 6). Risk factors associated with death were history of diabetes, dialysis type and time, unemployment, delayed graft function, visits to center, number of hospitalizations, and number of days in hospital. After multivariable analysis only time on dialysis, visits to center, and days in hospital were still associated with death (Table 7).

**Table 6:** Distribution of the causes of death over the 5 years of follow up

Period (months)	0-3	4-6	7-12	13-24	25-36	37-48	49-60	Total
Patients at risk	162	118	102	91	67	43	20	
Deaths, n (%)	40	16	15	24	24	23	20	162
Cause, n (%)								
Infection	20 (50)	9 (56)	9 (60)	9 (38)	16 (67)	10 (44)	12 (60)	85 (53)
Cardiovascular	14 (35)	6 (38)	2 (13)	7 (29)	2 (8)	5 (21)	4 (20)	40 (24)
Hemorrhagic shock	5 (13)	0 (0)	1 (7)	1 (4)	1 (4)	0	0	8 (5)
Malignant neoplasm	0	0	1 (7)	3 (12)	1 (4)	4 (18)	1 (5)	10 (6)
Nervous system	0	0	1 (7)		1 (4)	1 (4)	0	3 (2)
Undetermined	1 (2)	1 (6)	1 (7)	4 (17)	3 (13)	3 (13)	3 (15)	16 (10)

**Table 7:** Risk factors associated with death during the 5 years of follow up

Variables	Univariable analysis		Multivariable analysis	
	Hazard Ratio (95% CI)	*p*	Hazard Ratio (95% CI)	*p*
Recipient age, per year	1.001 (0.988 - 1.013)	0.943		
Recipient with history of diabetes	1.473 (1.049 - 2.068)	0.025	1.058 (0.734 - 1.526)	0.763
Renal replacement therapy				
Preemptive (ref)				
Hemodialysis	4.515 (1.118 - 18.227)	0.034	2.177 (0.524 - 9,040)	0.284
Peritoneal	6.028 (1.369 - 26.534)	0.018	4.348 (0.972 - 19.456)	0.055
Dialysis time, months	1.008 (1.004 - 1.012)	< 0.001	1.005 (1.001 - 1.009)	0.019
Education				
Secondary or higher (ref)				
Secondary incomplete or lower	1.339 (0.961 - 1.865)	0.085		
Profession				
Employed (ref)				
Unemployed	1.609 (1.182 - 2.190)	0.003	1.340 (0.966 - 1.858)	0.079
Distance to center	1.000 (0.999 - 1.000)	0.324		
Donor age	1.002 (0.999 - 1.014)	0.790		
Donor type				
Living (ref)				
Deceased	1.023 (0.704 - 1.487)	0.905		
Delayed graft function, yes	1.473 (1.077 - 2.014)	0.015	1.029 (0.730 - 1.451)	0.868
Treated acute rejection, yes	1.246 (0.907 - 1.713)	0.175		
Number of visits to the center, visits/month	1.743 (1.568 - 1.938)	< 0.001	1.750 (1.574 - 1.946)	< 0.001
Hospitalizations, yes	2.046 (1.315 - 3.184)	0.002	1.527 (0.947 - 2.463)	0.083
Days in hospital, days/month	1.011 (1.007 - 1.014)	< 0.001	1.015 (1.011 - 1.018)	< 0.001

## DISCUSSION

In this cohort of 1873 adult recipients of first kidney transplant, the 5-year patient (91.4%), graft (82.9%), and death-censored graft (90.7%) survivals are in agreement with other larger registry analyses.^9^^,^ 10 This case control study showed that infection is the most prevalent cause of death during the first 5 years after transplantation. Remarkably, 25% of all deaths occurred during the first 3 months after transplantation, a period of higher risk of mortality compared with patients on dialysis.^11^^,^ 12 At the end of the first year, 44% of all deaths had occurred. Cardiovascular disease was the second most prevalent cause of death in the sample. However, most of these deaths (64.3%) were in the first year post-transplantation, characterizing the high cardiovascular risk of the patients before transplantation.^13^ In the United States^14^ and Australia^15^ the main cause of death is cardiovascular disease, followed by infection and malignancy. Yet, in developing countries, the leading cause of death following renal transplant is infectious, followed by cardiovascular.^16^^-^ 18 The low incidence of death due to malignancy is perhaps associated with the still limited follow-up time of 5 years.

The difference in the primary cause of death is due to a complex interplay of donor, recipient and environmental factors. Time on dialysis is associated with increased risk and severity of infections, cardiovascular diseases, and malnutrition, which are comorbidities known to be associated with death after transplant.^19^^,^ 20 *Diabetes mellitus* is a well-known demographic characteristic associated with increased risk of mortality after kidney transplantation.^14^^,^ 21 The overall prevalence of recipients with medical history of *diabetes mellitus* was 24% with higher prevalence among patients who died during the 5 years of follow up. While a similar prevalence of 23% is observed in Europe,^22^ in the USA this prevalence is as high as 40%.^23^

The combination of inadequate deceased donor maintenance, the use of kidneys from expanded criteria, and the long cold ischemia time are known risk factors associated with the observed high incidence of delayed graft function. While a meta-analysis showed no significant relationship between delayed graft function and patient survival at 5 years,^24^ more recent registry analyses have shown an influence on long-term mortality.^25^ Furthermore, patients who develop delayed graft function are at higher incidence for acute rejection,^26^ inferior graft function,^27^^-^ 29 and patient survival.^27^^,^ 30 ^,^ 31

Patients who died had a higher prevalence of hospitalizations, hospitalization density, and visits to the transplant center than patients in the control group, perhaps due to higher number of comorbidities, complications after the transplant surgery, and worse transplant outcome. Hospitalizations are six times higher among kidney transplant recipients than the general population.^32^ While hospitalizations due to cardiovascular and infectious diseases are associated with higher mortality rate in the general population ,^33^ there is no such evidence among kidney transplant recipients.^32^^,^ 33

Sociodemographic characteristics of the transplant population such as education, profession, income, and development index are associated with transplant outcomes.^34^ Lower income was identified as a factor related to poor graft and patient survival in the United States.^4^ Woodward *et al*. showed that even in the first 3 years after transplant, when Medicare guarantees access to immunosuppression in the United States, patients with lower income present lower patient and graft survival.^4^ Also, access to health care is another key variable influencing transplant outcomes,^4^^,^ 35as evidenced when comparing 5-10 survivals in the USA and Europe.^35^ Limitations in access to health care and medication, with consequent negative influence on adherence to treatment, are key drivers of this observation. Despite the fact that access to health care is universal and free of charge in Brazil, patients with lower income share other difficulties such as financial burden with transportation to attend appointments and seek prompt care and purchase of concomitant drugs that are not provided by the government. Also, lack of health-related knowledge is associated with difficulties in understanding the beneficial effects of a balanced diet, physical activity, and adherence to treatment.^4^^,^ 36 ^,^ 37

Mortality in the general population is associated with HDI.^38^ Interestingly, HDI has also been correlated with transplant rates across countries.^6^ Remarkably, more than 95% of the patients included in this analysis were living in cities with high or very high HDI, with no difference between groups. Yet, the HDI of a city does not capture disparities within cities, such as highly developed regions surrounded by areas of significant poverty.

This analysis has limitations, including the single center case-control retrospective design using a relatively small cohort. By using a case-control design, important risk factors may have been hidden. The influence of immunosuppression could not be ascertained due to the relative homogeneity of the protocols. Given the wide geographical disparities of Brazil, interpretation and extrapolation of our results to other regions requires caution.

## CONCLUSION

In summary, this analysis confirmed that infection is the leading cause of mortality over the first 5 years after kidney transplantation. Several demographic and socio-economic risk factors were associated with death, most of which are not readily modifiable. Strategies to reduce mortality should include improvements in education, socioeconomic status, awareness and access to healthy habits including food and physical activity, increased social support, and easier access to healthcare.

## Abbreviations

BLSD: Blood and lymphatic system disorders
BPAR: Biopsy proven acute rejection
CD: Cardiac disorders
CMV: Cytomegalovirus
CTCAE: Common terminology criteria for adverse events
DGF: Delay graft function
DE: Endocrine disorders
eGRF: Estimated glomerular filtration rate
GD: Gastrointestinal disorders
GDASC: General disorders and administration site condition
HD: Hepatobiliary disorders
HDI: Human development index
HLA: Human leukocyte antigen
I: Investigation
IID: Infections and infestation disorders
IPPC: Injury, poisoning and procedural complications
MCTD: Musculoskeletal and connective tissue disorder
MND: Metabolism and nutrition disorders
mTor: Mammalian target of rapamycin
NBMU: Neoplasms benign, malignant and unspecified (incl cysts and polyps)
NS: Nervous system
NS-SEC: National socio-economic classification
PD: Psychiatric disorders
r-ATG: Rabbit antithymocyte globulin
RTMD: Respiratory, thoracic and mediastinal disorders
RUD: Renal and urinary disorders
SAE: Serious adverse event
VD: Vascular disorder
